# An Epitome of the History of Medicine

**Published:** 1899-07

**Authors:** 


AN EPITOME OF THE HISTORY OF MEDICINE. By ROSWELL PARK, A. M., M. D., Professor of Surgery in the Medical Department of the University of Buffalo, etc. Based upon a course of lectures delivered in the University of Buffalo. Second edition. Illustrated. Pp. xiv.—370. Philadelphia: The F. A. Davis, Co., Publishers. 1899.

In the issue of the JOURNAL for February, 1898, was reviewed the first edition of Dr. Park's History of Medicine, and urged that it should be in the possession of every physician and especially every young doctor. No doubt this advice has been partially heeded, for within a year from the publication of the first edition, a second has been called for.

No great changes have been made in this edition, except the addition of a chapter on iatrotheurgic symbolism, which was originally read by invitation before the Maine Medical Association in June, 1898.

In appearance and make-up the volume is similar to the first edition.

